# The effectiveness and efficiency of diabetes screening in Ontario, Canada: a population-based cohort study

**DOI:** 10.1186/1471-2458-10-506

**Published:** 2010-08-20

**Authors:** Sarah E Wilson, Laura C Rosella, Lorraine L Lipscombe, Douglas G Manuel

**Affiliations:** 1 Institute for Clinical Evaluative Sciences, 2075 Bayview Avenue, Toronto, Ontario, Canada; 2 Dalla Lana School of Public Health, University of Toronto, 155 College Street, Toronto, Ontario, Canada; 3 Women's College Hospital, 76 Grenville Street, Toronto, Ontario, Canada; 4 Department of Medicine, University of Toronto, Toronto, Ontario, Canada; 5 Ottawa Hospital Research Institute, 725 Parkdale Avenue, Ottawa Ontario, Canada; 6 Statistics Canada, R.H. Coats Building 24 A, 100 Tunney's Pasture, Ottawa, Canada

## Abstract

**Background:**

Little is known about the efficiency and effectiveness of the current level of diabetes screening activity in Ontario where there is universal access to health services. Our study aims were to: (i) determine how often Ontarians are screened for diabetes; (ii) estimate screening efficiency based on the number needed to screen (NNS) to diagnosis one diabetes case; (iii) examine the population effectiveness of screening as estimated by the number of undiagnosed diabetes cases.

**Methods:**

Ontario respondents of the Canadian Community Health Survey who agreed to have their responses linked to health care data (n = 37,400) provided the cohort. The five-year probabilities of glucose testing and diabetes diagnoses were estimated using a Cox Proportional Hazards Model. We defined NNS as the ratio of diabetes tests to number of diabetes diagnoses over the study period. We estimated the number of undiagnosed diabetes by dividing the number not tested at the end of study period by the NNS.

**Results:**

80% of women and 66% of men had a blood glucose test within 5 years. The efficiency of screening was estimated by a NNS of 14 among men and 22 among women. 127,100 cases of undiagnosed diabetes were estimated, representing 1.4% of the Ontario adult population. Increasing age, hypertension, immigrant and non-white ethnicity, and number of general practitioner visits were associated with an increased likelihood of having a glucose test (LR χ2 p < 0.001). Low income men were less likely to be tested.

**Conclusions:**

Diabetes screening was high in this population-based cohort of Ontarians. Screening efficiency varied considerably in the population. Undiagnosed diabetes continues to be prevalent and remains concentrated in the highest risk groups for diabetes, especially among men.

## Background

The global burden of diabetes is increasing and has been well documented [1-3]. In addition to its large disease burden, evidence suggests that one-third to one-half of all diabetes cases continue to be 'undiagnosed' in industrialized settings [4-9]. These estimates come from population health surveys that compare physiologic measures [fasting blood glucose or oral glucose tolerance test results] with respondents' self-report of diabetes. These surveys are resource intensive and as a consequence, few countries implement them on an ongoing basis with some exceptions [4,5].

Type 2 diabetes (diabetes) can remain asymptomatic for up to 10 years [10], and at the time of diagnosis 20-30% will have already developed complications [11,12]. Early detection and treatment of diabetes through screening has been proposed to prevent or slow the development of microvascular and macrovascular complications [13,14]. In Canada there is no formal diabetes screening program or policy. However, two clinical guidelines provide directions for screening. Since 1998, the Canadian Diabetes Association (CDA) has recommended screening individuals aged 45 years and older every three years with a fasting blood glucose test, and earlier and/or more frequently for individuals with risk factors [15-17]. The age to initiate screening was reduced to 40 in the 2003 CDA guidelines [16]. These recommendations were predominantly consensus-driven, due to insufficient evidence of direct benefits from screening (Grade D). The Canadian Task Force on Preventive Health Care (CTFPHC) [18] recommends screening only for adults with established hypertension or hyperlipidemia, for whom benefits of early diabetes detection and treatment have been shown.

Although glucose testing has increased significantly among Ontarians since these guidelines were implemented [19], little is known about the efficiency and effectiveness of the current level of diabetes screening activity in Ontario where there is universal access to health services. Estimates on the burden of undiagnosed diabetes in Canada are extrapolated from data from other industrialized countries.

Using population-based Ontario databases, our objectives were to (i) determine how often people are screened for diabetes in the overall Ontario population and in different at-risk groups; (ii) estimate Ontario's screening efficiency based on the number needed to screen (NNS) to diagnose one case of diabetes; and (iii) examine the population effectiveness of screening as estimated by the number of Ontarians with undiagnosed diabetes.

## Methods

### Cohort definition

Ontario respondents of the 2000/2001 Canadian Community Health Survey (CCHS, Cycle 1.1) [20] who agreed to have their data linked to Ontario Health Insurance Plan (OHIP) billing data provided the base cohort. Ninety-one percent of Ontario respondents agreed to have their data linked (n = 37,400). Ontario is Canada's most populated province with an ethnically diverse population of 12.7 million in 2006 [21]. The CCHS is a representative national survey administered by Statistics Canada on an annual basis to collect information on self-perceived health, chronic conditions, socio-demographic and socio-economic information on community dwelling adults and adolescents aged 12 and over [20]. Responding to the survey is voluntary and data are collected directly from survey respondents. Those living on Indian Reserves and Crown Lands, institutional residents, full-time members of the Canadian Armed Forces, and residents of certain remote areas are excluded from the sampling frame. The CCHS uses the area frame of Canada's Labour Force Survey for sampling which excludes persons living on reserves and other Aboriginal settlements in the provinces, full-time members of the Canadian Armed Forces and the institutionalized population [22]. The survey uses a multistage stratified cluster design and provides cross-sectional data representative of 98% of the Canadian population over the age of 12 years and attained a national response rate of 84.7% for the 2000/2001 cycle [20].

Individuals were excluded if, at the time of survey completion (September 1, 2000 to November 30, 2001), they were less than 20 years of age, had a physician diagnosis of diabetes defined by inclusion in the Ontario Diabetes Database [23], self-reported pregnancy, or were ineligible for OHIP services at any point in the 12 months prior to survey completion. The Ontario Diabetes Database is a cumulative diabetes registry that uses administrative health records to determine diabetes status. A detailed description of its methodology can be found elsewhere [23].

### Data sources

After our cohort was created using the CCHS survey database, data for each individual were linked to administrative health databases that include records for all individuals eligible for health services under the government-funded Ontario Health Insurance Plan (OHIP), using unique encrypted health card numbers. Individual-level data from each Ontario CCHS respondent in the cohort was deterministically linked to individual-level information on health services accessed through OHIP and to other administrative health databases. The OHIP database was used to identify laboratory service claims for diabetes testing. All Ontario residents are eligible for coverage by OHIP after 3 months of residency in the province. Legislation prohibits the private delivery of services covered under OHIP, including laboratory testing.

The diabetes status of respondents was established by linking individuals to the Ontario Diabetes Database (ODD), which contains all patients said to have physician-diagnosed diabetes identified since 1991 and their date of diagnosis on the basis of administrative health data. An individual is said to have physician-diagnosed diabetes if at least one of the following criteria are met: (i) hospital admission with a diabetes diagnosis; (ii) a physician services claim with a diabetes diagnosis followed within 24 months by either a further physician service claim or a hospital admission with a diabetes diagnosis. A hospital record with a diagnosis or pregnancy care or delivery close to a diabetic record (eg 90 days before and 120 days after the diabetes record date) is considered to relate to gestational diabetes and is not included in the ODD. The ODD has been validated against primary-care records and demonstrated to be accurate for determining the incidence and prevalence of diabetes (sensitivity 86%, specificity 97%) [23,24]. Eligibility for OHIP health services and information on deaths was captured from the Registered Person Database (RPD) and self-reported demographic information was derived from the CCHS. Data from April 1, 2001 to March 31, 2006 were extracted for analysis. All data extraction and analyses were carried out using SAS (Version 9.1).

### Baseline variables of interest

Information on socio-demographic characteristics and self-reported health states was available from the CCHS. These variables included age, sex, urban/rural address, income quartiles, education quartiles, ethnicity, immigrant status, current smoking, self-reported heart disease, self-reported hypertension and derived body mass index (BMI) from self-reported weight and height. A binary variable was derived to indicate whether an individual met the CDA recommendations for screening. If an individual met any one of the following criteria: age > 40, non-white ethnicity, self reported heart disease, or self-reported hypertension, they were considered to be meet the CDA recommendations for screening. Using the OHIP database, the number of general practitioner/family physician (GP/FP) visits and the number of specialist visits (non-primary care providers) that occurred in the twelve months prior to survey completion was determined for each individual. The 5-year risk of developing diabetes was determined using the Diabetes Population Risk Tool (DPoRT) aggregated into deciles of risk. DPoRT is a validated population-based risk tool developed in Ontario to predict incidence of physician-diagnosed diabetes for up to a 10-year time period using the variables in the CCHS [25,26]. The predictive factors included in the DPoRT algorithm are BMI, age, ethnicity, hypertension, immigrant status, smoking, education status and heart disease [25,26]. The algorithm has high discrimination (C = 0.8) and accuracy and has been validated in 2 external populations including the cohort used in this study. A separate publication describes the development and validation of this population-level risk algorithm in greater detail [26].

### Survival analysis

Members of the study cohort were deterministically linked to OHIP fee codes for serum blood glucose (SBG) laboratory tests using unique encrypted health card numbers (OHIP codes G002, L111, L112) [27]. No OHIP fee code discriminates between fasting and random serum blood glucose measurement. We have previously demonstrated that the SBG is the most common laboratory test used to identify diabetes in Ontario, representing 87% of all diabetes-related laboratory tests (among SBG, HbA1c, and oral glucose tolerance tests) undergone by individuals without a pre-existing physician-diagnosis of diabetes in the year 2005 [19]. We have also previously demonstrated that oral glucose tolerance tests are rarely used in Ontario with fewer than 2500 tests ordered in the year 2005 among an adult population size exceeding 9 million [19]. Survival analysis was selected as the analytic approach to investigate diabetes testing over a five year time period, and at the same time examine diabetes incidence in the same cohort. Respondents were followed from the time of their individual CCHS 1.1 survey completion date (September 1, 2000-November 30, 2001) until their first SBG test, diabetes diagnosis date or end of study period (March 31, 2006). Diabetes diagnosis was defined by date of entry into the ODD. This date is the same as the date of an individual's contact with the health system that met the criteria for diabetes diagnosis under the ODD algorithm. The following events were used to right censor further individual data: a date of death as recorded in the RPDB, and cessation of OHIP eligibility during the observation period as defined by no OHIP eligibility for a full fiscal year. The midpoint of the fiscal year (October 1) was used as the time point when eligibility was considered to have ended. Time to SBG testing was estimated using a Cox Proportional Hazards Model. All estimates were calculated using bootstrap survey weights to accurately reflect the demographics of the Ontario population.

### Measures of efficiency and population effectiveness of diabetes screening

We defined the efficiency of screening as the ratio of the number of screening tests for diabetes to the number of diabetes diagnoses. We used the term 'number needed to screen' (NNS) to represent screening efficiency. To compute 'number needed to screen' the number of individuals tested with a SBG over the five year study period was divided by the number of incident diabetes cases that accrued during the same period (NNS = number of individuals tested with at least one SBG test over 5 years/the number of individuals with new diabetes diagnoses over 5 years). The purpose of this term is to capture the efficiency of testing in different at-risk groups. We defined the population effectiveness of screening as the magnitude of undiagnosed cases of diabetes at the end of the study period. The number of individuals with undiagnosed diabetes was estimated by dividing the number of individuals not tested with a SBG at the end of five years by the 'number needed to screen' (number with undiagnosed diabetes = N - number of individuals tested by 5 years/NNS). The measure of 'undiagnosed diabetes' is an estimate of how many additional cases of diabetes would be detected in a scenario of 100% screening. Both NNS and the number of 'undiagnosed' diabetes cases were calculated for each covariate, and its varying levels of risk.

### Research ethics

Ethics approval was granted from the institutional review board at Sunnybrook Health Sciences Centre, Toronto, Ontario, Canada.

## Results

### Rates and Predictors of glucose testing

The baseline characteristics of our cohort are presented in Table 1. At the end of the five-year study period, over 80% of women and 66% of men underwent a SBG test between 2001 and 2006 (Table 2). In adults aged 40 and over, 84% of women and 63% of men underwent diabetes testing.

**Table 1:** Baseline characteristics of the cohort.

	Males	Females
Characteristic	(N = 11,684)	(N = 14,103)
BMI (mean/median)	26.1/25.6	24.9/24.0
Age (mean/median)	44/42	46/44
		
Age <45, %	56.5	51.9
45≤Age < 65, %	30.8	31.6
Age≥65, %	12.6	16.6
		
BMI < 23	22.2	39.5
23≤BMI < 25	21.5	17.8
25≤BMI < 30	40.0	27.2
30≤BMI < 35	12.8	9.4
BMI≥35	3.0	4.0
		
Non-white, %	16.5	16.2
Immigrant, %	30.0	29.6
Hypertension, %	12.0	14.4
Current Smoker, %	24.9	19.1
Heart Disease, %	4.9	5.0
Graduated Post Secondary School, %	61.8	58.7
Low Income, %	5.9	9.0
Rural Residence, %	15.4	14.1
		
> = 1 Specialist visit year prior, %	33.5	48.2
Median GP visits year prior	2.0	4.0
CDA recommended testing	71.9	73.3
		
5-year diabetes incidence rate, %	4.7	3.7
SBG Test in 5 years	66.1	80.6
		

**Table 2:** Univariate comparisons of diabetes risk factors and screening, diabetes incidence, and yield of screening

	Males					Females				
	Overall N	SBG Screening Rates N (%)	Diabetes Incidence Rate N (%)	NNS	Undiagnosed N (%)	Overall N	SBG Screening Rates N (%)	Diabetes Incidence Rate N (%)	NNS	Undiagnosed N (%)
**Age**										
< 30	770,046	338,031 (43.9)	3,775 (0.5)	90	4,813 (0.64)	724,622	530,460 (73.2)	9,258 (1.3)	57	3,389 (0.47)
30-40	932,346	549,356 (58.9)	17,516 (1.9)	31	12,211 (1.31)	878,512	678,356 (77.2)	23,993 (2.7)	28	7,079 (0.81)
40-50	895,685	622,058 (69.5)	37,893 (4.2)	16	16,668 (1.86)	915,611	738,888 (80.7)	26,571 (2.9)	28	6,355 (0.69)
50-60	592,437	488,119 (82.4)	54,358 (9.2)	9	11,617 (1.96)	605,545	527,646 (87.1)	33,802 (5.6)	16	4,990 (0.82)
60-70	352,317	301,598 (85.6)	34,063 (9.7)	9	5,728 (1.63)	425,580	367,952 (86.5)	24,965 (5.9)	15	3,910 (0.92)
70-80	237,503	200,328 (84.4)	27,067 (11.4)	7	5,023 (2.11)	326,074	284,792 (87.3)	24,579 (7.5)	12	3,563 (1.09)
> = 80	80,671	69,634 (86.3)	6,076 (7.5)	11	963 (1.19)	140,307	122,712 (87.5)	6,682 (4.8)	18	958 (0.68)
										
**BMI**										
< 23	858,877	543,837 (63.3)	17,909 (2.1)	30	10,375 (1.21)	1,585,528	1,224,805 (77.3)	22,181 (1.4)	55	6,533 (0.41)
23-25	830,834	537,390 (64.7)	25,365 (3.1)	21	13,851 (1.67)	715,910	589,129 (82.3)	10,862 (1.5)	54	2,338 (0.33)
25-30	1,545,276	1,051,697 (68.1)	63,206 (4.1)	17	29,664 (1.92)	1,093,914	910,076 (83.2)	56,542 (5.2)	16	11,422 (1.04)
30-35	492,792	335,215 (68.0)	51,068 (10.4)	7	24,006 (4.87)	375,544	318,462 (84.8)	31,460 (8.4)	10	5,639 (1.50)
≥35	117,323	90,112 (76.8)	21,361 (18.2)	4	6,450 (5.50)	159,092	136, 950 (86.1)	18,327 (11.5)	7	2,963 (1.86)
										
**Urban**										
Yes	595,749	2,199,153 (62.1)	156,774 (4.8)	14	15,103 (0.63)	3,449,416	2,803,812 (81.3)	130,476 (3.4)	21	30,043 (0.87)
No	3,265,256	369,972 (67.4)	23,973 (4.0)	15	23,973 (3.24)	566,835	447,001 (78.9)	19,374 (3.8)	23	5,194 (0.92)
										
**Income**										
Medium - High Income	3,307,677	2,212,001 (66.9)	151,223 (4.6)	15	74,906 (2.26)	3,274,131	2,660,077 (81.2)	114,373 (3.5)	23	26,411 (0.81)
Low Income	226,796	150,533 (66.4)	14,931 (6.6)	10	7,564 (3.34)	362,139	292,618 (80.8)	17,314 (4.8)	17	4,113 (1.14)
Missing income	326,533	206,591 (63.3)	14,592 (4.5)	14	8,472 (2.59)	379,781	298,118 (78.5)	18,164 (4.8)	16	4,976 (1.31)
										
**Education (%)**										
< Than Post-Secondary	1,463,580	953,241 (65.1)	85,215 (5.8)	11	46,108 (3.14)	2,345,860	1367352 (82.9)	81,053 (4.9)	17	16,733 (1.01)
Post-Secondary Graduation	2,370,510	1,596,255 (67.3)	94,249 (4.0)	17	46,330 (1.95)	1,649,628	1867019 (79.6)	68, 453 (2.9)	27	17,556 (0.75)
										
**Ethnicity**										
White	3,221,837	2.089,112 (64.8)	138,556 (4.3)	15	75,126 (2.33)	3,364,092	2,697,355 (80.2)	125,615 (3.7)	21	31,050 (0.92)
Non-white	634,399	471,945 (75.2)	40,345 (6.4)	12	13,460 (2.14)	649,657	552,197 (85.0)	24,134 (3.7)	23	4,255 (0.66)
										
**Immigrant**										
Yes	1,158,710	895,326 (77.3)	76,424 (6.6)	12	22,482 (1.94)	1,188,590	1,027,063 (86.4)	52,249 (4.4)	20	8,217 (0.69)
No	2,300,910	1,672,413 (61.9)	103,751 (3.8)	16	63,805 (2.36)	2,825,492	2221610 (78.6)	97,421 (3.5)	23	26,481 (0.94)
										
**CDA recommended testing**										
Yes	2,776,919	2,066,820 (74.4)	174,532 (6.3)	12	59,964 (2.16)	2,943,259	2,471,320 (84.0)	137,070 (4.7)	18	26,176 (0.89)
No	1,084,087	502,305 (46.3)	6,216 (0.6)	81	7,200 (0.66)	1,072,992	779,493 (72.7)	12,380 (1.2)	63	4,661 (0.43)
										
**Hypertension**										
Yes	464,040	398,018 (85.8)	52,367 (11.3)	8	8,686 (1.87)	578,863	517,314 (89.4)	51,606 (8.9)	10	6,140 (1.06)
No	3,398,054	2,168,513 (64.0)	128,373 (3.8)	17	72,361 (2.13)	3,433,812	2,729,924 (79.5)	98,245 (2.9)	28	25,332 (0.74)
										
**Has Heart Disease**										
Yes	190,236	158,9 67 (83.6)	23,433 (12.3)	7	4,609 (2.42)	198,884	176,103 (88.6)	10,431 (5.2)	17	1,349 (0.68)
No	3,670,118	2,409,746 (65.7)	157,314 (4.3)	15	8,228 (0.32)	3,816,102	3,073,446 (80.5)	139,305 (3.7)	22	33,661 (0.88)
										
**GP visits year prior**										
0	1,106,642	563,932 (51.0)	38,156 (3.5)	15	36,720 (3.32)	595,001	398,240 (66.9)	14,285 (2.4)	28	7,058 (1.19)
1 to 3	880,234	601,531 (68.3)	36,891 (4.2)	16	17,092 (1.94)	881,194	707,051 (80.2)	22,589 (2.6)	31	5,564 (0.63)
3 to 6	643,173	507,004 (78.8)	36,911 (5.7)	14	9,913 (1.54)	922,366	772,054 (83.7)	42,438 (4.6)	18	8,262 (0.90)
more than 6	610,842	517,983 (84.8)	54,679 (9.0)	9	9,802 (1.60)	1,144,539	1,012,660 (88.5)	59,031 (5.2)	17	7,688 (0.67)
										
**Any specialist visits year prior**										
Yes	1,291,364	1,012,569 (78.4)	82,866 (6.4)	12	22,816 (1.77)	1,935,924	1,656,767 (85.6)	85,879 (4.4)	19	14,470 (0.75)
No	2,569,642	1,556,555 (60.6)	97,882 (3.8)	16	63,707 (2.48)	2,080,327	1,594,047 (76.6)	63,971 (3.1)	25	19,515 (0.94)
										
**Current Smoker**										
Yes	960,272	562,180 (58.5)	30,296 (3.2)	19	21,453 (2.23)	765,308	573,968 (75.0)	27,488 (3.6)	21	9,163 (1.20)
No	2,899,597	2,006,504 (69.2)	150,452 (5.2)	13	66,966 (2.31)	3,248,525	2,675,254 (82.4)	122,362 (3.8)	22	26,220 (0.81)
										
**Deciles of 5-year Diabetes Risk**										
1	550,105	169,194 (55.4)	4,887 (0.89)	62	6,144 (1.12)	464,029	340,733 (73.4)	1,424 (0.31)	239	516 (0.11)
2	329,707	242,041 (51.3)	2,134 (0.65)	79	1,110 (0.34)	391,905	284,318 (72.5)	2,587 (0.66)	110	978 (0.25)
3	477,327	256,151 (50.7)	2,680 (0.56)	90	2,458 (0.51)	433,997	337,126 (77.7)	4,609 (1.1)	73	1,327 (0.31)
4	430,093	263,288 (59.6)	5,483 (1.3)	47	3,549 (0.83)	568,618	440,384 (77.4)	10,316 (1.8)	43	2,982 (0.52)
5	348,449	254,219 (75.6)	13,566 (3.9)	19	4,959 (1.42)	375,633	297,289 (79.1)	12,192 (3.3)	24	3,264 (0.87)
6	383,153	292,415 (66.3)	17,797 (4.6)	14	6,481 (1.69)	414,068	352,650 (85.2)	12,568 (3.0)	28	2,194 (0.53)
7	379,107	254,447 (77.1)	18,386 (4.9)	16	7,791 (2.06)	306,102	252,960 (82.6)	17,859 (5.8)	14	3,796 (1.24)
8	317,279	247,118 (80.2)	22,045 (7.0)	12	5,847 (1.85)	372,234	325,893 (87.6)	22,011 (5.9)	15	3,089 (0.83)
9	314,169	269,534 (78.7)	30,238 (9.6)	8	5,579 (1.78)	325,268	275,806 (84.8)	24,374 (7.5)	11	4,497 (1.38)
10	331,619	169,194 (81.3)	63,531 (19.2)	4	40,606 (12.24)	364,398	327,766 (89.9)	41,911 (11.5)	8	4,579 (1.26)
										
**Overall**	3,861,006	2,569,124 (66.5)	180,747 (4.7)	14	92,277 (2.39)	4,016,251	3,250,814(80.9)	149,850 (3.7)	22	34,793 (0.87)

Among women, the groups observed to have the highest rates of testing were those in the highest (tenth decile) diabetes risk group (89.9%), those with self-reported hypertension (89.4%), self-reported heart disease (88.6%) and those with more than six primary care visits in the year preceding CCHS survey completion (88.5%). Among men, the highest rates of testing were observed in men aged 80 and over (86.3%), those with self-reported hypertension (85.8%), and those with more than six primary care visits in the year prior to the survey (84.8%).

Among women, increasing age, increasing BMI, hypertension, immigrant status, non-white ethnicity, and number of GP visits were all associated with a significantly increased likelihood of having a SBG test in the multivariate survival model (LR χ2 p < 0.001) (Table 3). Among men, the same factors were significantly associated with an increased likelihood of undergoing a SBG test, with the exception of increasing BMI which was at the margins of significance (adjusted HR 1.01, 95% CI 1.00-1.02). Men with low incomes were significantly less likely to undergo SBG testing (adjusted HR 0.86, 95% CI 0.75-0.98). Among women, those who did not self-report income were significantly less likely to undergo an SBG (adjusted HR 0.88, 95% CI 0.79-0.98) while low income women were no more or less likely to undergo testing. The probability of SBG testing increased with age for both men and women, but increasing age appeared to be a more powerful determinant of SBG testing for men (Table 3).

**Table 3:** Unadjusted and adjusted hazard ratios and 95% (bootstrapped) confidence intervals by sex

	MALES				FEMALES			
	UNADJUSTED		ADJUSTED		UNADJUSTED		ADJUSTED	
Variable	Hazard Ratio	95% CI	Hazard Ratio	95% CI	Hazard Ratio	95% CI	Hazard Ratio	95% CI
Age								
< 30	1.00	ref	1.00	ref	1.00	ref	1.00	ref
30-40	1.53	(1.33, 1.77)	1.41	(1.22, 1.63)	1.17	(1.07, 1.29)	1.12	(1.02, 1.24)
40-50	2.08	(1.82, 2.37)	1.93	(1.69, 2.21)	1.34	(1.22, 1.47)	1.32	(1.19, 1.46)
50-60	3.24	(2.82, 3.71)	2.82	(2.46, 3.24)	1.77	(1.60, 1.96)	1.65	(1.48, 1.83)
60-70	4.19	(3.62, 4.86)	3.43	(2.95, 3.98)	1.80	(1.61, 2.01)	1.64	(1.46, 1.84)
70-80	4.20	(3.54, 4.98)	3.13	(2.61, 3.77)	2.07	(1.86, 2.30)	1.81	(1.62, 2.03)
> = 80	4.70	(3.85, 5.74)	3.19	(2.56, 3.98)	2.22	(1.92, 2.58)	1.86	(1.54, 2.26)
								
BMI (continuous)	1.01	(1.01, 1.02)	1.01	(1.00, 1.02)	1.02	(1.02,1.03)	1.01	(1.01, 1.02)
								
Hypertension (Yes)	2.12	(1.95, 2.32)	1.27	(1.16, 1.39)	1.59	(1.49,1.70)	1.12	(1.03, 1.22)
								
Income								
Med-High	1.00	ref	1.00	ref	1.00	ref	1.00	ref
Low	0.96	(0.87, 1.14)	0.86	(0.75, 0.98)	1.02	(0.93, 1.13)	0.92	(0.84, 1.02)
Not Stated	0.93	(0.79, 1.08)	0.93	(0.80, 1.08)	0.94	(0.84, 1.06)	0.88	(0.79, 0.98)
								
Immigrant	1.56	(1.43, 1.70)	1.26	(1.15, 1.37)	1.38	(1.28, 1.48)	1.24	(1.15, 1.34)
								
Non-white ethnicity	1.34	(1.20, 1.50)	1.38	(1.23, 1.55)	1.23	(1.11,1.36)	1.19	(1.06, 1.33)
								
Number of GP visits year prior								
0	1.00	ref	1.00	ref	1.00	ref	1.00	ref
1-3	1.47	(1.35,1.59)	1.42	(1.31, 1.55)	1.31	(1.19, 1.44)	1.31	(1.19, 1.44)
3-6	2.02	(1.85, 2.20)	1.72	(1.57, 1.87)	1.53	(1.42, 1.67)	1.52	(1.40, 1.65)
> = 6	2.86	(2.59, 3.17)	2.13	(1.90, 2.38)	1.96	(1.80, 2.13)	1.81	(1.66, 1.98)

**Figure 1:**
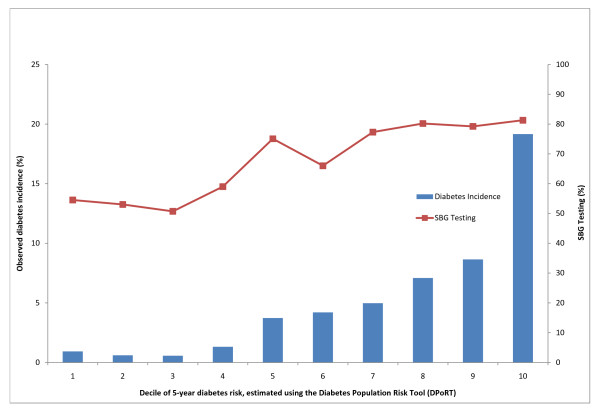
**SBG testing (%) and observed diabetes incidence (%) by decile of five-year diabetes risk for males from 2001-2006**.

**Figure 2:**
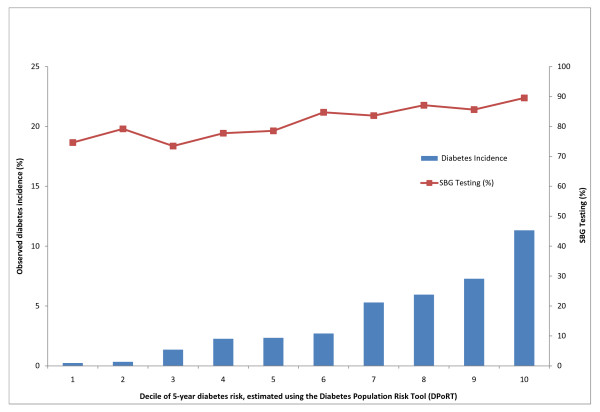
**SBG testing (%) and observed diabetes incidence (%) by decile of five-year diabetes risk for females from 2001-2006**.

Figures 1 and 2 depict the relationship between SBG testing and the five-year risk of diabetes for men and women. In both sexes there is a tendency towards greater observed testing with increased risk of diabetes with higher rates of screening observed among women, across all deciles of diabetes risk.

### Efficiency of Diabetes Testing

Upon conclusion of the five year study period, the observed incidence rate of diabetes was 4.7% among men and 3.7% among women in this cohort (Table 1, Table 2). In men, the highest diabetes incidence rates were observed among those with the highest (tenth decile) predicted diabetes risk (19.2%), those with a BMI >35 (18.2%), self-reported heart disease (12.3%), those age 70-80 years (11.4%), and self-reported hypertension (11.3%). Similar findings were seen in women (Table 2).

We used the summary statistic of 'number needed to screen' (NNS) to capture the efficiency of screening in different groups. Only 14 men and 22 women required screening in order to detect an incident case of diabetes respectively, in the overall Ontario population. The highest yield of testing was seen among those with a BMI > 35, where only 4 men and 7 women needed to be tested in order to yield one new diagnosis of diabetes. Diabetes testing was much less efficient in other risk groups, notably younger women who appeared to be tested at a rate disproportionate to their risk of diabetes. The highest NNS value in our study at 239 was observed for women in the lowest decile of diabetes of which 77% underwent testing between 2001 and 2006. Women with other low risk attributes including having a BMI less than 23, age under 30, and women who the CDA would not recommend screening, were also observed to have a testing rate of 77% at the end of the study period. Table 2 displays the variation in the NNS estimates both within and between different sub-groups. For example, there was little difference in the NNS estimates for some subgroups such as urban residence as evidenced by a NNS of 14 for urban and a NNS of 15 for non-urban men; ethnicity with an observed NNS of 21 for white women and a NNS of 23 for women of non-white ethnicity; and similar NNS estimates for self-reported immigrant and non-immigrant status within each gender. In contrast, there were other subgroups where the differences in NNS were more apparent within the subgroup such as BMI, self-reported hypertension, self-reported heart disease and the summary variable indicating whether the CDA would recommend testing. For example, the NNS estimate for men recommended to be screened by the CDA is 12, in contrast to a NNS of 81 for men not recommended to be screened in accordance with the CDA guidelines

### Population Effectiveness of Diabetes Testing

At the end of the study, we estimated 1.4% of Ontario adults (127,100 cases) had undiagnosed diabetes. Among the male cohort, 2.4% were estimated to have undiagnosed diabetes comprising three-quarters (92,300) of the total number of undiagnosed cases. Among men, the most common groups remaining with undetected diabetes were those in the tenth decile of risk (12.2%), men with BMIs > 35 (5.5%) and BMIs 30-35 (4.9%), low income (3.3%), and those with no GP visits in the year preceding CCHS survey completion (3.3%). Among women, there were an estimated 34,800 cases of undiagnosed diabetes at the end of the study period (0.9% of all women). The groups with the highest percentage of undiagnosed diabetes among females were those with BMI >35 (1.9%), BMI 30-35 (1.5%), ninth decile of risk for diabetes (1.4%), those refusing to self-report income (1.4%), and tenth decile of risk for diabetes (1.3%).

**Figure 3:**
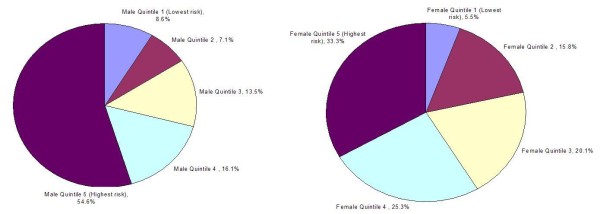
**Distribution of undiagnosed diabetes by quintile of diabetes risk for men and women**.

We found that groups with high rates of screening still maintained high proportions of undetected diabetes. Men in the tenth decile of diabetes risk had the highest percentage of undiagnosed diabetes at 12.2% despite a screening rate of 81%. The highest rate of undiagnosed diabetes among women was found among those with BMIs >35 at 1.9%, despite an 86% screening rate. Figure 3 displays the distribution of undiagnosed cases of diabetes by quintile of diabetes risk. Among men, 53% of all undiagnosed diabetes is estimated to occur among men in the highest quintile of diabetes risk. Due to the higher rates of screening observed among women, only one-third of undiagnosed diabetes is found in the highest quintile of diabetes risk with greater representation of undiagnosed diabetes in lower quintiles of risk.

## Discussion

This population-based study documents a high degree of serum blood glucose testing in the community-dwelling population of Ontario adults without diabetes. Between 2001 and 2006, 80% of women and 66% of men aged 20 and older underwent glucose testing. In addition, persons with risk factors for diabetes, such as older age, high BMI, hypertension and non-white ethnicity, were appropriately tested significantly more often. Screening in Ontario appears to be largely efficient, with a yield of testing that results in 14 men and 22 women requiring glucose testing to yield one new diagnosis of diabetes, respectively. Glucose testing is most efficient for groups with elevated BMIs with NNS values as low as 4, and least efficient for low risk women who appear to undergo a disproportionate amount of testing relative to their risk of diabetes. Despite high rates of testing in higher risk groups, undiagnosed diabetes remains prevalent, especially among men wherein three-quarters of all undiagnosed diabetes is found. The highest rates of undiagnosed diabetes were found among those in the highest deciles of diabetes risk, those with elevated BMIs, and among men, low-income status, despite testing rates as high as 80%.

Despite high levels of screening and a publicly funded health care system with universal access, an estimated 28% of all diabetes in the province of Ontario remains undiagnosed. This compares similarly with the results of the most recent NHANES surveys in the United States where the percentage of undiagnosed diabetes was found to be 30.1% in the 1999-2002 survey [4] and 34.6% in the 1988-1994 survey [5]. In Ontario, men are more likely to be undiagnosed than women. This reflects both a lower rate of testing and a higher incidence of diabetes in men relative to women. Barriers to undergoing diabetes testing can be presumed to operate at the level of the individual, clinic and the health system. In our multivariate analysis we found that men with low incomes and women who did not self-report income were significantly less likely to under SBG testing, after controlling for other variables, including BMI. These findings suggest that not only is low income status a risk factor diabetes [28], but it appears also to be a risk factor for having undiagnosed diabetes, even in a system with universal access to publicly funded health care services. This suggests that a strategy beyond the dissemination of clinical practice guidelines for diabetes testing is required to address undiagnosed diabetes in low income populations.

The analyses presented here have several implications for diabetes screening. This study shows that, in general, clinicians' screening behaviour increases in proportion to the risk of diabetes, with increasing proportions of individuals tested with increasing 5-year risk of diabetes. However, at the extremes of risk there appears to be a mis-match between screening and diabetes risk. Low-risk individuals, in particular women, undergo a disproportionate amount of screening in relation to their low-risk status resulting in large estimates for the NNS, while the screening rates of higher-risk individuals could be increased further, in order to further reduce the burden of undiagnosed diabetes. This finding could be a reflection of a healthy user effect. However, it is important to note that our methodology for the conceptualization of efficiency, NNS, may be over-estimated in lower risk individuals and be under-estimated in higher-risk individuals owing to the fact that the ODD is used for a determination of diabetes status and is likely to have a greater false positive rate associated with low risk individuals due to the lower prevalence of diabetes. We are not aware of any previous publication that has documented the population-level rates of diabetes screening to which our data can be compared; however, some studies have reported on the frequency and yield of testing within a single health care organization [29]. Our data compare similarly with a retrospective study from a large Health Maintenance Organization which found a screening rate of 73% among women and 66% among men after three years [29]. However, this study examined screening only among adults aged >45 years, for whom both the CDA and the American Diabetes Association recommend routine testing. Our data examined diabetes screening in adults over the age of 20, which includes a large number of adults that typically would not be recommended to undergo diabetes screening, unless significant risk factors are present. We found that the rates of screening in Ontario among those aged 40 and over were relatively unchanged from our overall estimates, with 84% of women and 63% of men tested between 2001 and 2006.

Other investigators have conceptualized NNS in the same way and have utilized the measure to examine the yield of diabetes screening in Japan [30]. However, the yield of screening was examined for subjects presenting for a periodic health examination within a hospital setting, rather than a population based analysis [30]. The NNS values found in that study were 16 for men and 32 for women. In our population-based cohort of adult Ontarians we found that 14 men and 22 women required screening in order to yield one incident diagnosis of diabetes, respectively. The concept of 'number needed to be screened' was originally developed by Rembold in 1998 to define a summary statistic analogous to the 'number needed to treat' that would allow for the comparison of different screening strategies on mortality reduction [31]. Rembold used mortality data from randomized controlled trials (RCTs) of screening interventions and defined number needed to screen as "the number of people that need to be screened to prevent one death". Given the lack of RCT in the area of diabetes screening, and the paucity of evidence demonstrating that earlier detection of diabetes results in mortality reduction [18] we instead defined NNS in relation to the yield of incident diabetes diagnoses over a five-year period.

Most of what is currently known about the prevalence of undiagnosed diabetes (screening effectiveness) comes from data collected as part of cross-sectional population health surveys which compare biological markers, such as blood glucose testing, with self-reported health states including diabetes. Here we demonstrate that surveillance of 'undiagnosed' diabetes can be accomplished with the use of longitudinal population-based administrative health data that include information on testing and diabetes diagnoses. This analytic approach allows current screening practices to be evaluated from the three perspectives of population coverage, efficiency and effectiveness. Surveys with biologic markers typically report undiagnosed DM as a proportion of total cases of diabetes (diagnosed and undiagnosed). The diabetes database used in our study provides population estimates of diabetes diagnosis [24], which can be combined with the findings of this study to report undiagnosed diabetes cases as a proportion of the estimated total of diabetes in Ontario. Among men, the estimated 92,300 cases of undiagnosed diabetes represent 16% of the total burden of diabetes over the same time period (563,000 cases of prevalent and newly diagnosed cases). Among women, there were an estimated 34,800 cases of undiagnosed diabetes at the end of the study period, comprising 7.8% of the total burden of diabetes (443,000 cases of prevalent and newly diagnosed diabetes).

Surveillance of diabetes is a necessary first step towards its prevention and control [1,32]. However, there are many challenges associated with surveillance of diabetes and other chronic diseases that fall outside of traditional surveillance mechanisms that currently exist for communicable diseases and cancer [32]. Novel models of diabetes surveillance are being developed, including the New York City Department of Health and Mental Hygiene's A1C Registry which was initiated in 2006 [33]. However, even with creative techniques the capture of undiagnosed diabetes remains a major challenge to robust diabetes surveillance. The analytic approach presented here allows for an estimation of undiagnosed diabetes, in addition to other important population attributes of current screening practices with the use of administrative health data sources alone.

The strengths of our study include the use of population-based administrative data linked to a population health survey to examine the time to first serum blood glucose test in a cohort of community dwelling adults in Ontario. Individuals with diabetes were identified and removed from our analysis with the use of a validated diabetes database [24] and laboratory data were linked to individuals with a unique identifier. However, there are some important limitations that deserve mention. First, we were not able to differentiate between fasting and random serum blood glucose tests using our data. Since only fasting blood glucose testing is recommended for the diagnosis of diabetes in asymptomatic individuals, our analysis provides an estimation of the degree of screening for diabetes. Other investigators examining diabetes screening have found that serum blood glucose is the most common test ordered for screening [19,29]. Due to our use of administrative health data (OHIP billings) to estimate diabetes case detection, our data captures testing in asymptomatic individuals [screening] as well as testing in symptomatic individuals (diagnosis). However, given that most individuals are asymptomatic at the time of diabetes diagnosis [11], the majority of testing captured in this study is likely to represent opportunistic screening. Second, our estimates of diabetes were derived from a registry that is associated with a 14% false negative rate, which confers some risk of misclassification. Third, although we have captured all diabetes-related laboratory services billed to OHIP, we were not able to capture lab tests carried out within the global budget of hospital inpatient services. However, this limitation at most resulted in an underestimate of the current degree of diabetes testing in Ontario. Finally, the survival analysis methodology employed in this study involved following individuals until the date of respondents' first diabetes laboratory test or diabetes diagnosis. However, this limited our ability to examine the relationship between the frequency of testing and future diabetes risk. This is an important area for future investigations.

## Conclusions

Diabetes screening in Ontario is common with screening rates of 80% among women and 66% among men over a five year time period, and is largely efficient. The NNS, our measure of screening efficiency, is 14 for men and among women is 21 which likely reflects that clinicians are appropriately testing individuals with risk factors for diabetes and that diabetes testing is easily accomplished with a simple, readily available and inexpensive test. Screening is least efficient in low-risk women who are commonly screened, and screened disproportionately in relation to many moderate and high risk groups. Despite high rates of screening in Ontario, undiagnosed diabetes among men remains clustered among those at highest risk for diabetes suggesting a disproportionately low amount of testing in relation to risk. Furthermore, low income men are less likely to undergo testing. To further improve the overall population effectiveness of screening, higher risk individuals may need specific targeting which may require programs that seek to reduce the barriers to accessing traditional screening in primary care and other traditional health care settings.

## Abbreviations

BMI: Body mass index; CCHS: Canadian Community Health Survey; CDA: Canadian Diabetes Association; FPG: Fasting plasma glucose; HbA1c: Hemoglobin A1c; ODD: Ontario Diabetes Database; OGTT: Oral glucose tolerance test; OHIP: Ontario Health Insurance Plan; RPDB: Registered Persons Database; SBG: Serum blood glucose.

## Competing interests

The authors declare that they have no competing interests.

## Authors' contributions

SEW and DGM formulated the study's design. LCR was responsible for data acquisition and analysis. SEW drafted the original manuscript. SEW, LLL, LCR, and DGM made substantial contributions to the analysis and interpretation of data and revised the manuscript for important intellectual content. All authors read and approved the final manuscript.

## Pre-publication history

The pre-publication history for this paper can be accessed here:

http://www.biomedcentral.com/1471-2458/10/506/prepub
